# Observe, Practice, and Improve? Enhancing Sidestep Cutting Execution in Talented Female Soccer Players: A Four-Week Intervention Program With Video Instruction

**DOI:** 10.1519/JSC.0000000000004796

**Published:** 2024-04-25

**Authors:** Eline M. Nijmeijer, Matthias Kempe, Marije T. Elferink-Gemser, Anne Benjaminse

**Affiliations:** Department of Human Movement Sciences, University Medical Center Groningen, University of Groningen, Groningen, the Netherlands

**Keywords:** motor learning, observational learning, injury prevention, biomechanics

## Abstract

Nijmeijer, EM, Kempe, M, Elferink-Gemser, MT, and Benjaminse A. Observe, practice and improve? Enhancing sidestep cutting (SSC) execution in talented female soccer players: A four-week intervention program with video instruction. *J Strength Cond Res* 38(8): e430–e439, 2024—Implicit learning has the potential to improve movement execution and reduce injury risk. Previous research showed beneficial effects of short-term interventions with implicit learning in male athletes. However, research on long-term interventions in female athletes is lacking. The aim of this study was to examine the effects of a 4-week intervention with video instruction on movement execution of SSC, a task that is highly related with anterior cruciate ligament (ACL) injury risk, in female athletes. Twenty talented adolescent female soccer players were part of the control (CTRL, *n* = 10) or video instruction (VIDEO, *n* = 10) group. All subjects practiced 4 weeks and received general task instructions. In addition, the VIDEO group received expert video instruction during practice. Lower extremity kinematics and kinetics and vertical ground reaction force of SSC were examined during baseline, immediate post, and 1-week retention tests. After nonlinear registration, differences between each subject and the expert she had seen were determined. These differences were analyzed with SPM1D 2-way ANOVA. No interaction effects between time and group were found (*p* > 0.05). Main effects of time were found in the frontal plane. In particular, smaller deviations of subjects compared with the seen expert of the knee adduction (*p* = 0.005, 97.9–100% stance phase [SP]) and hip abduction (*p* = 0.005, 11.5–13.8% SP) and adduction (*p* < 0.001, 33.4–87.7% SP) moments were found in immediate post compared with baseline. These frontal plane short-term improvements, replicating earlier findings in both sexes, may lower ACL injury risk. The large observed interindividual differences over time may have concealed the long-term effects of video instruction at the group level.

## Abstract 2

Supplemental Digital Content is available in the text.

## Introduction

The rising incidence of anterior cruciate ligament (ACL) injury in young athletes is alarming (3,55). Studies reported a 20% rate of a second ACL injury (2,58) and an even higher rate for young athletes (<20 years) (53) or those who return to the preinjury level (32). Female athletes are more prone to rupture their ACL compared with male athletes in soccer (10). Approximately half of all ACL ruptures occurs with a noncontact mechanism (33). Prospective evidence regarding risk factors of ACL injuries in sidestep cutting (SSC) is lacking. Moreover, as SSC movement executions in laboratory and field differ from each other (17,18), we cannot fully transfer potential risk factors in laboratory to field. However, specific biomechanical risk factors related to noncontact ACL injuries on field such as increased knee abduction, hip abduction, and external rotation of the knee were observed in video analyses (33). These variables may therefore be related to ACL injury. Because biomechanical risk factors are modifiable, changing movement execution has the potential to mitigate injury risk (5,16,38). The rate of ACL injuries in young female athletes is still increasing (55), and the effectiveness of female ACL injury prevention programs has been questioned (4,6,7). Therefore, enhanced understanding of how we can help female athletes learn to optimize sport-specific movements is needed. To modify movement execution, principles from the domain of motor learning (e.g., implicit learning and self-controlled practice) could be used to reduce high-risk knee loading and potentially injury risk (4,61).

One way to learn motor skills is through implicit learning. Implicit learning focuses on the outcome or the goal of the movement, requires little working memory, and is characterized by a high degree of automaticity (34). By contrast, explicit learning focuses on the details of movement execution and specific knowledge about the performance of a skill (35). Athletes who learned skills implicitly perform more stable (i.e., less errors, more robust) under stress, fatigue, or other environmental factors compared with athletes who learned skills explicitly (6,9). Learning skills implicitly can be stimulated with (video) observations or analogies. With observations, athletes can extract important information and requirements of the task without specific cues or instructions. The athlete will explore and then select the movement solution that fits best in his/her own body (6). This “whole-body approach” enhances being embedded in the task (embodied cognition) and seems to be an effective method to promote motor learning (4). Alternating observing (during rest) and physically practising a task has been shown to enhance retention and transfer (61) and could therefore be the basis of a prevention program.

Recent research has broadened our understanding of observation learning for enhancing motor skill acquisition. The review article by Ste-Marie et al. (50) summarized the literature about observational learning and outlined several important features (*when, who, what, and how*) that need to be considered when designing observation interventions. First, it has been shown that observation before or during the practice period is most effective (*when*). Second, although there is still a debate regarding which model type is optimal, model similarity is important resulting in the advice to use peer models, i.e., matching anthropometrics and sex (4,13) (*who*). Third, no difference between provisions of live vs. video modeling has been found. The choice could be made based on feasibility (*what*). Regarding the *how*, no consensus exists regarding the viewing angle and the speed of video demonstration (50). However, showing videos from behind (posterior view, same perspective as practising) has been shown to lead to improvements in cutting (5) and jumping execution (13). In summary, live demonstration or videos from peers from a posterior view with high model similarity (anthropometrics and sex) should be incorporated before or during training when designing observation interventions.

Of the 48 articles included in the review by Ste-Marie et al. (50), solely 2 articles examined the effects of observational learning using video instruction on the movement execution of a sport-specific task in healthy athletes (8,45). In addition, a more recent short-term intervention study showed improved movement execution after providing video instruction (39). The beneficial effects (i.e., improved power clean lift and cutting execution) showed the potential of video instruction on movement execution (8,45). On the other hand, the ample research performed points to the lack of knowledge about the applicability of using video instruction in complex sport-specific tasks (i.e., sidestep cutting) that are related to ACL injuries. Some 6-week technique modification programs have been evaluated in, for example, American Football (51) or multidirectional sports (20). These interventions primarily focused on deceleration, SSC, and agility exercises performed in subgroups, while the control groups continued their normal training program. These studies showed the potential that intervention programs aimed to improve movement execution could have on the risk of ACL injury. Research has shown differences in motor learning (5) and differences in movement executions (44,46) between sexes. However, no study has specifically looked at the effect of video instruction on changing movement executions in female athletes in the long-term (i.e., multiple week interventions and including a retention test).

The evidence discussed above demonstrates the potential that observational learning can have on improving movement execution. However, it is also apparent that little research has examined this potential in female athletes in sport-specific tasks. To potentially improve prevention programs, the aim of this study was to examine the effects of a 4-week intervention with video instruction on movement execution of SSC, a task that is highly related with ACL injury risk, in female athletes. It is hypothesized that video instruction helps to improve learning, and therefore, better movement execution is expected after the intervention.

## Methods

### Experimental Approach to the Problem

A controlled 4-week intervention study with a repeated measures design was used. Two groups were used: a video intervention (VIDEO) and control (CTRL) group. Each subject practiced for 4 weeks (training phase). The program included (a) unanticipated SSC, (b) single-leg jumping and landing, and (c) double-leg jumping and landing (each 10 trials) to offer a variety of movements all related to injury prevention. Furthermore, a baseline test before the first training session, immediate post-test directly after the last training session, and a 1-week retention test (a basic version) of the tasks (testing phase) were performed. The measurements were performed at the same time of the day for each subject to control for circadian rhythm. Three-dimensional motion and ground reaction force analysis were used to examine the intervention's effectiveness.

### Subjects

No power analysis was performed before this study, as there is no software available yet to perform 1D (i.e., time series data) sample size estimation for 2-way analyses. Therefore, we based our sample size on previous studies within biomechanical research (12,19,51). Twenty healthy talented female soccer players (mean age 14.9 ± 1.0 years, height 168.2 ± 5.1 cm, mass 56.1 ± 7.3 kg) of Regional Talent Center Soccer North (Groningen, The Netherlands) participated (Table 1). The first cohort of players formed the VIDEO group; the second cohort of players formed the CTRL group. The subjects trained 4 times a week with a mean duration of 75 minutes per training and played 1 match per week. For inclusion, subjects had to be between 12 and 18 years. Potential subjects were excluded if (a) they are injured to the lower extremity, (b) have/had other relevant injuries or surgery, or (c) had a history of neurological, vestibular, or visual impairments.

**Table 1:** Anthropometric data of subjects (mean ± *SD*).

	CTRL (*n* = 10)	VIDEO (*n* = 10)	*p*
Age (y)	14.4 ± 0.3	15.3 ± 1.2	0.016
Height (cm)	168.3 ± 5.5	168.1 ± 5.0	0.990
Mass (kg)	55.0 ± 7.8	57.2 ± 6.9	0.513

### Procedures

Ethical approval was obtained from the University Medical Center Groningen (ID No. METc 2018.249). Written informed consent of subjects and legal guardians was obtained before inclusion. First, 16 reflective markers of 14 mm in diameter according to the Vicon Plug-in-Gait lower-body model (Vicon Motion Systems, Inc., Centennial, CO) were placed. Five additional trunk markers on the sternum, clavicle, C7, T10, and right scapula were attached, as a commonly used set-up, designed for practical application, in this type of research (5,39,41). Body height and mass, leg length, knee, and ankle width were measured and entered into the Vicon Nexus Software (version 2.10) to make an accurate biomechanical model. This was followed by a static calibration consisting of standing still for 3 seconds in a T-pose on 2 Bertec force plates (Bertec Corporation, Columbus, OH) (52).

Subjects were allowed to choose 1 of the 3 packages (order of tasks) (a) 1-2-3, (b) 2-3-1, and (c) 3-1-2 before the training session started. Subjects had to choose a different order of the tasks each week. As the weeks progressed, the tasks increased in complexity using an increase of visual, coordinative, and physical load; this included changes of location, time, speed, and direction. This progression was embedded in the tasks, while the core and goal of the tasks remained unchanged. Furthermore, competition elements (i.e., scoring a goal) were added to increase sport-specificity and motivation. In addition, contextual interference and with this, increased sport-specificity, more possible (unanticipated) directions, increasing parkour length, and passing balls at targets were added to all tasks during the weeks. All tasks were performed with a sports buddy, who was part of the research group, with the aim to increase enjoyment, motivation, and sport-specificity (unanticipated element) during the training. All subjects knew this and were instructed that the sports buddy served for this purpose only (i.e., not to copy movement of sports buddy). A detailed description of the tasks can be seen in the Supplemental Digital Content 1 (see http://links.lww.com/JSCR/A486).

#### Task

**Figure 1.**
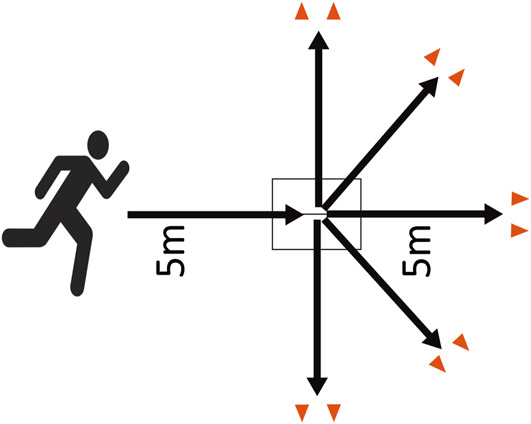
Unanticipated sidestep cutting (SSC) task.

The unanticipated SSC task was analyzed in this paper as it is the most complex task of the intervention and highly related to ACL injury risk. Subjects used a 5-m approach run followed by a 1-foot landing on the force plate and a 0° (i.e., running straight forward), 45°, or 90° change in the direction followed by running through a gate 5 m away from the force plates (Figure 1). A sports buddy was running in front of the subject and randomly selected a direction to run to. The sports buddy started 1.5 m in front of the subject, and as soon as the sports buddy started to run, the subject followed. The sports buddy was trained by AB before the data collection started and verbally coached throughout all sessions/trials to enhance consistency in approaching speed and cutting angles. A SSC trial was only included for the analysis if the sports buddy and subject made a cut angle of 45° toward the nondominant leg direction with the dominant leg on the force plate. Leg dominance was defined as the leg the subject preferred jumping and landing with (5,39). A 45° toward the nondominant leg direction was chosen as this angle is frequently investigated in research related to ACL injuries (5,20,38,52).

#### Apparatus

**Figure 2.**
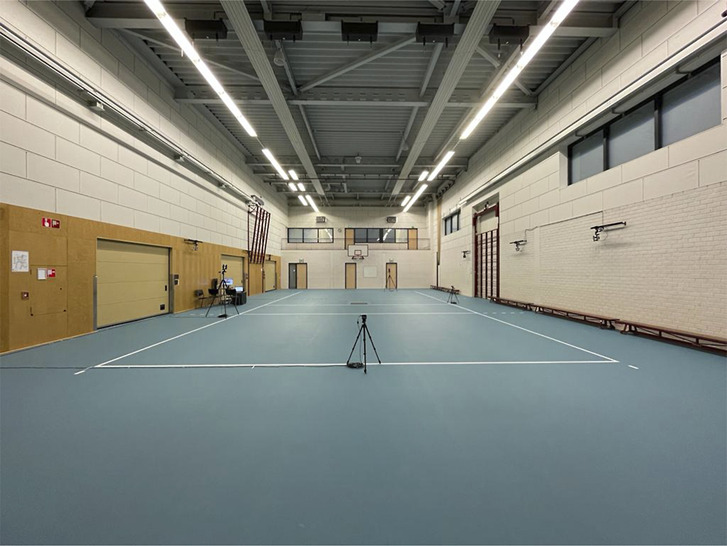
Apparatus setup; 6 Vicon Vantage V5 cameras mounted on the wall, 2 Vicon Vantage V5 cameras on tripods (obliquely in front and back), 2 Vicon Vue HD cameras on tripods (sagittal and posterior), and 2 Bertec force plates embedded in the floor.

Lower-body kinematics and vertical ground reaction force (vGRF) data were captured using a 100 Hz 8-camera motion analysis system (Vicon Motion Systems, Inc.), Vicon Nexus Software (version 2.10 Motions Systems, Inc.), and 2 1,000 Hz Bertec force plates (Bertec Corporation). High test and retest repeatability and good measurement accuracy of Vicon motion analysis have been reported previously (27,36). After intensive training by a senior researcher (AB), AB and EN attached the markers during the sessions. Therefore, we do expect a high inter-reliability and intrareliability regarding the sensor placement. Two 50 Hz Basler video cameras (cameras with a 25- and 9-mm C-mount lens, Basler Inc., Exton, PA) were used, one filming the sagittal plane from a lateral view and the other filming the frontal plane from a posterior view (Figure 2).

#### Collecting Expert Data

Expert videos of the included tasks were obtained before the experimental study started to provide video instruction to the subjects. Three talented female soccer players (a) 170 cm, 52 kg; (b) 174 cm, 62 kg; and (c) 177 cm, 58 kg, who were not subjects, served as expert models and were peers from the subjects. These players from Regional Talent Center Soccer North (Groningen, The Netherlands) were selected by coaches and deemed to have excellent motor skills at this stage. They came individually to the laboratory and were instructed by AB to perform 45° toward the nondominant leg direction SSC movements using previously reported figures (16) and verbal instructions. The experts were encouraged to find a way to imitate the shown figures as best as possible and performed 10 to 15 trials. Selection of the expert videos was based on the movement technique, following the same method, as previously described (39,56). Details about the kinetics and kinematics of expert trials are shown in a table in Supplemental Digital Content 2 (see http://links.lww.com/JSCR/A487). In short, the experts showed a preferable movement exeuction with alignment of hip and knee in the intended direction. Moreover, the knee flexion angle is highly increased from initial contact (IC) to weight acceptance and the body is aligned with the vGRF vector.

#### Collecting Subject Data

Each subject was matched to 1 of the 3 experts based on height. After receiving the general instruction and goal by the investigator (AB or EN), each subject performed 3 to 5 familiarization trials before performing the 10 trials which were used for the data analysis. Before the first trial, subjects in the VIDEO group watched expert videos performing the task. Subjects stood straight behind the laptop to stimulate a realistic feeling and whole-body awareness (embodied cognition). The 2 videos were randomly presented from the posterior frontal and sagittal sides on a laptop and played at circa 70% of real speed (38). To stimulate implicit motor learning, subjects were instructed by the investigator (AB or EN) to *“Please have a look at the entire movement of the expert. When making the turn, try to imagine how this feels and try to imitate the expert to the best of your ability”* (39,56). No additional verbal instructions pinpointing at specific body parts were given. During the training sessions, the subjects of the VIDEO group watched the same videos once again before the sixth trial. All subjects were given enough rest (at least 30 seconds) between the trials to reduce the potential effects of fatigue.

#### Data Processing

**Figure 3.**
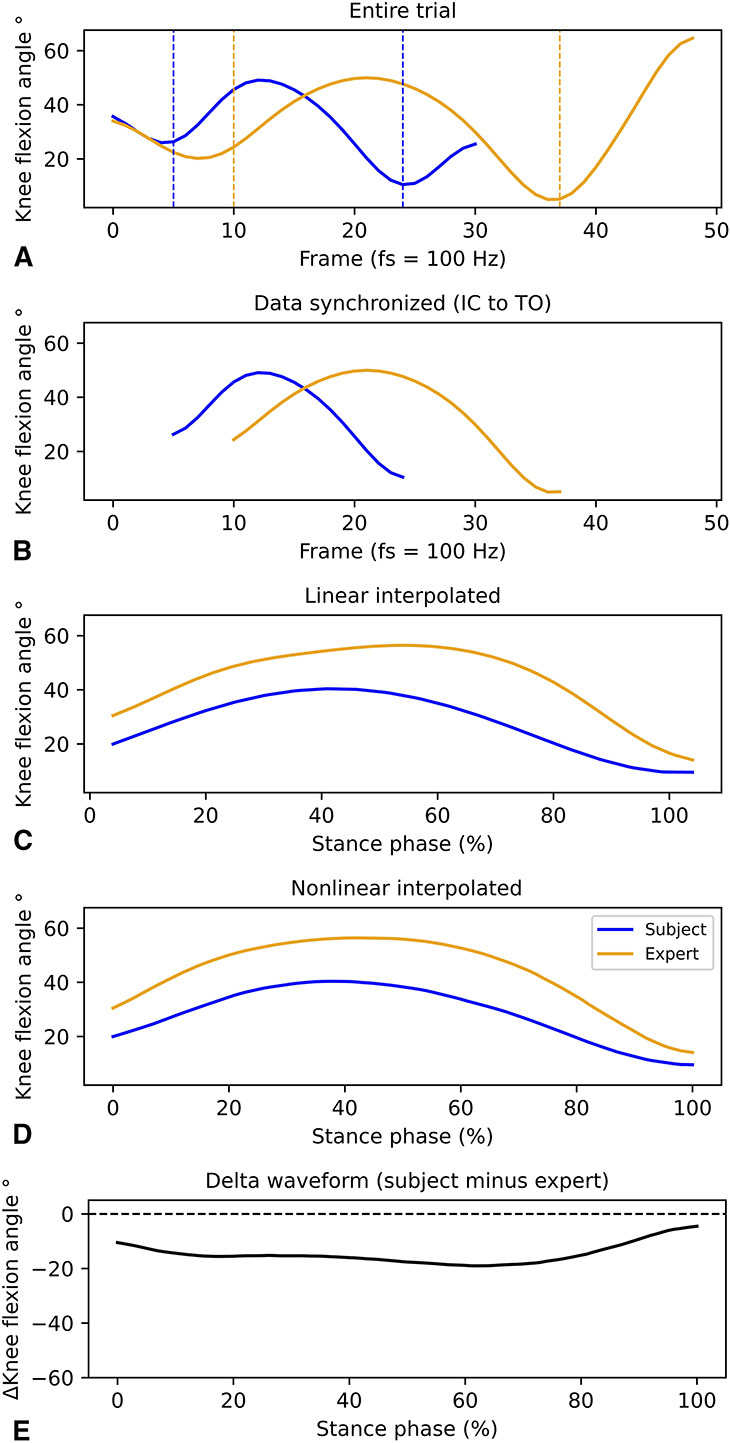
Graphical overview of processing data. Panel A shows waveform of expert trial and subject trial. Panel B shows cropped data based on synchronization kinematic and kinetic data. Stance phase (SP) was determined from initial contact (IC) (vertical ground reaction force [vGRF] > 20N) to toe-off (vGRF < 20N). Panel C shows linear interpolation for each trial to 101 data points with each point representing 1% of the SP. Panel D shows nonlinear registration of the data. Panel E shows delta waveforms which are calculated by subtracting the waveform of the expert from the waveform of the subject (i.e., positive values indicate greater degrees/moments of subject compared with expert).

Primary outcome measures were lower-body kinematics and kinetics (i.e., hip, knee, and ankle sagittal angles and moments and hip and knee frontal angles and moments) and vGRF. Using the pipeline function in Vicon Nexus Software, joint coordinate (marker) and force data were filtered using a zero-lag fourth-order low-pass Butterworth filter. As the literature has not reached consensus about the optimal cutoff frequency, i.e., some proposed matching frequencies (30), whereas others used different frequencies for marker and force data (20,41,54), a residual analysis (59) was used to determine the optimal cutoff frequency of every trial with the python package *optcutfreq* (22). After visual inspection, the mean optimal cutoff frequency was chosen, which was 10 and 125 Hz for marker and force data, respectively. A customized python script (Python Software Foundation, DE) was used to preprocess the data and perform statistical analysis. External moments and vGRF were normalized to body mass. The analysis used in this paper is based on work done by Pataky et al. (42). The steps taken to perform the final analysis are shown in Figure 3. Synchronization was based on the first instant of vGRF higher than 20 N (IC) to below 20 N (toe-off) (21). After that, each trial was first linearly interpolated to 101 data points and nonlinearly registered using the square-root slope framework (42). Nonlinear registration allows discriminating between (post hoc) amplitude and timing effects. In other words, after using this type of registration, analysis regarding amplitude differences between the 2 groups is more valid compared with simultaneously testing amplitude and timing effects (which is the result of testing linear registered data) (42). In this study, 3 expert videos were used, and not every subject saw the same video. To correct for this, delta waveforms were determined between the subject and the expert she had observed. Delta waveforms were calculated by subtracting the waveform of the expert from the waveform of the subject (i.e., positive values indicate greater angles/moments of subject compared with expert).

### Statistical Analysis

The open-source software package spm1D 0.4 (https://spm1d.org/) was used to perform Statistical Parametric Mapping (SPM) analyses. Since the normality assumption was violated shown with the D'Agostino-Pearson K2 normality tests (*p* < 0.05), nonparametric procedures were performed (43). To examine any differences at baseline, a nonparametric independent *t* test was performed between CTRL and VIDEO group with an alpha divided by 3 (*α* = 0.0167) to correct for multiple testing. To determine differences in landing movement executions, nonparametric 2-way ANOVA analysis with repeated measures on 1 factor (time) was performed to analyze group (CTRL and VIDEO) and time (baseline, immediate post, and retention) effects. For both analyses, the delta waveforms were used, meaning that the smaller the mean delta waveform was, the closer the subjects performed to the expert. As the current paper focuses on amplitude effects, the significance level was set a priori to <0.025 (42). When the waveform exceeded the critical threshold, the data were considered significantly different in that part of stance. In addition, a nonsphericity correction by adjusting the degrees of freedom was applied since the assumption of identical variance could not be justified. Nonparametric paired *t* tests with Bonferroni corrections (https://spm1d.org/doc/PostHoc/anova.html) were used as post hoc tests if significant different clusters (of time) within subjects were found. Since responses to ACL injury prevention programs can be very dissimilar in individuals (15,23,51), baseline to retention differences for each subject of each variable were determined. This was done by subtracting the delta waveform of retention from the delta waveform of baseline (i.e., higher values indicate a higher change from baseline to retention).

## Results

**Figure 4.**
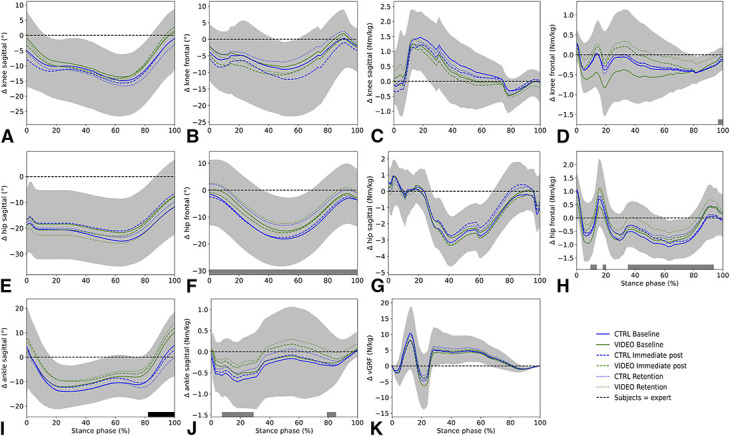
Mean delta waveforms and *SD* clouds of variables (0–100% stance phase [SP]). For readability, we just plotted the maximum SD (regardless of group and test). Gray and black bars at the bottom indicate clusters of significance for the main effect of test (gray) or main effect of group (black) (i.e., gray bar in plot F indicates a main effect of test during the complete SP). Please note; delta is depicted on the y-axis, and positive values indicate greater degrees/moments of subject compared with expert.

**Figure 5.**
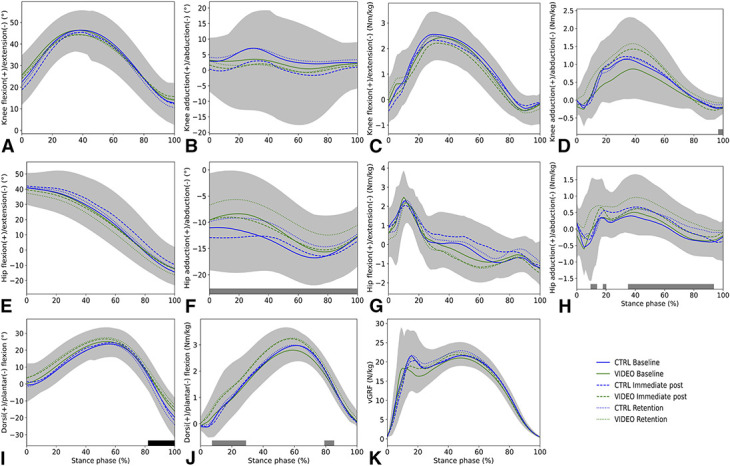
Mean waveforms and *SD* clouds of variables (0–100% stance phase [SP]). For readability, we just plotted the maximum *SD* (regardless of group and test). Gray and black bars at the bottom indicate clusters of significance for the main effect of test (gray) or main effect of group (black) (i.e., gray bar in plot F indicates a main effect of test during the complete SP).

**Figure 6.**
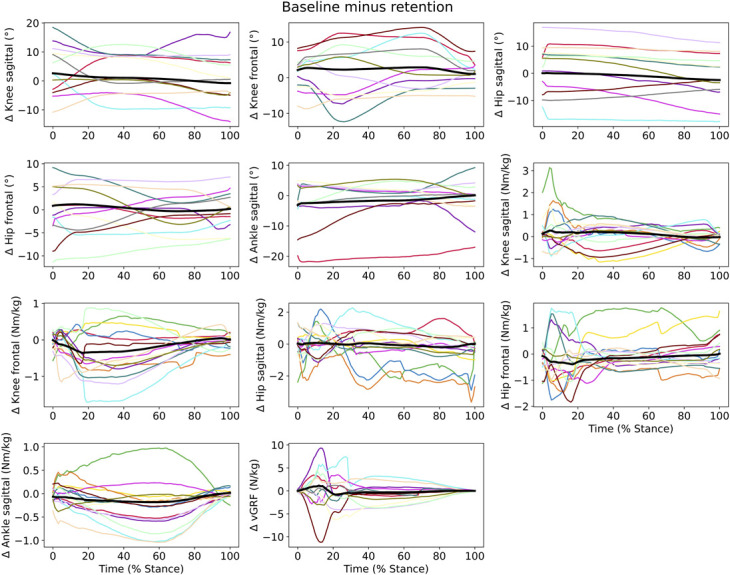
Individual baseline to retention changes of all variables. Greater values indicate a higher change from baseline to retention. Each color represents 1 subject. The black line indicates the mean change from baseline to retention.

No significant differences between the CTRL and VIDEO group existed at baseline (*p* > 0.0167), although baseline values differed largely between individuals (see Figure, Supplemental Digital Content 3, http://links.lww.com/JSCR/A488). No interaction effects between time and group were found for all variables (Table 2). Significant differences were found between the tests taking all subjects together (main effect of time factor). Hip abduction angle (*p* < 0.001), hip abduction moment (3 clusters; *p* = 0.013, *p* = 0.018 & *p* < 0.001), ankle plantar flexion moment (2 clusters; *p* = 0.005 & *p* = 0.015), and knee abduction moment (*p* = 0.004) significantly differed between baseline, immediate post, and retention. Post hoc comparisons showed smaller differences between subject and experts (lower Δ) of the hip abduction angle in immediate post compared with retention (*p* = 0.003, 1.7–22.3% stance phase [SP]) and smaller deviations of the knee adduction moment in immediate post compared with baseline (*p* = 0.005, 97.9–100% SP) and retention (*p* = 0.003, 96.3–100% SP). Moreover, subjects showed smaller deviations from experts regarding hip abduction (*p* = 0.005, 11.5–13.8% SP) and adduction moments (*p* < 0.001, 33.4–87.7% SP) in immediate post compared with baseline. Greater ankle plantar flexion angles were found in VIDEO compared with CTRL (*p* = 0.002, 82.9–100%). Figure 4 shows the mean delta waveforms and maximum SD clouds; Figure 5 shows the mean waveforms and maximum SD clouds (without comparison with the experts). Individual baseline to retention differences are shown in Figure 6.

**Table 2:** *p*-values (% of stance phase) of 2-way ANOVA with repeated measures on time and post hoc analysis.*

	Main effect time	Main effect group	Interaction effect time*group	Post hoc
				Time
Δ Hip flexion angle (°)	n.s.	n.s.	n.s.	n.s.
Δ Hip abduction angle (°)	<0.001 (0–100%)	n.s.	n.s.	Imm < ret: 0.003 (1.7–22.3%)
Δ Knee flexion angle (°)	n.s.	n.s.	n.s.	n.s.
Δ Knee abduction angle (°)	n.s.	n.s.	n.s.	n.s.
Δ Ankle plantar flexion angle (°)	n.s.	0.002 (82.9–100%) VIDEO > CTRL	n.s.	n.s.
Δ Hip flexion moment (Nm·kg^‒1^)	n.s.	n.s.	n.s.	n.s.
Δ Hip abduction moment (Nm·kg^‒1^)	0.013 (9.3–14.0%)	n.s	n.s.	Base vs. imm: 0.005 (11.5–13.8%) and <0.001 (33.4–87.7%)
	0.018 (17.6–20.4%)			
	<0.001 (34.6–94.0%)			
Δ Knee flexion moment (Nm·kg^‒1^)	n.s.	n.s.	n.s.	n.s.
Δ Knee abduction moment (Nm·kg^‒1^)	0.004 (96.5–100%)	n.s.	n.s.	Base > imm: 0.005 (97.9–100%)
				Ret > imm: 0.003 (96.3–100%)
Δ Ankle plantar flexion moment (Nm·kg^‒1^)	0.005 (7.3–28.9%)	n.s.	n.s.	n.s.
	0.015 (78.4–85.5%)			
Δ vGRF (N·kg^‒1^)	n.s.	n.s.	n.s.	n.s.

* Δ = delta indicating difference between subjects and expert; SP = stance phase; vGRF = vertical ground reaction force; base: baseline test; imm = immediate post-test; ret = retention test.

## Discussion

The aim of this study was to examine the effects of a 4-week intervention with video instruction on movement execution of SSC, a task that is highly related with ACL injury risk, in female athletes. It was hypothesized that video instruction enhanced learning, and therefore, better movement execution was expected after the intervention in the VIDEO group compared with the CTRL group. The hypothesis is rejected as no interaction effects were found between the 2 groups. At the same time, the results implicate that the subjects, regardless of group, improved their execution at the immediate post-test compared with baseline. In more detail, subjects showed hip and knee abduction moments closer to the expert during immediate post compared with baseline. Low hip and knee abduction moments are linked to a more optimal knee joint loading (14,33). These improvements in execution partly disappeared 1 week after training (retention test) as the deviation of subjects compared with experts regarding knee abduction moment and hip abduction angle increased from immediate post to retention. Learning takes time and consolidates normally over at least 1 night of sleep (28,47). The intervention seemed to have led to short-term but not to long-term adaptations in some variables.

Previous studies found mixed effects of cutting modification programs and video interventions. Six-week training programs were effective in changing cutting execution in male soccer players (19,51). Moreover, 2 training sessions with video instructions achieved retention of improved cutting (8) and jumping (56,57) execution. Training with video feedback was sufficient to improve cutting execution in male, but not in female basketball players (5).

In contrast to these studies (8,19,51), we did not find long-term adaptations; several suggestions could be given as an explanation. First, the subjects in this study were quite experienced. Most of the previous research was conducted in novices, and not the same effects could be expected. Experienced athletes may need more training to enhance as there is less room for improvement (15). The processes involved in changing movement executions differ from learning a new skill, and thus, appropriate approaches for experienced athletes should be considered to successfully modify already existing skills (48). Moreover, evidence for female youth is scarce (11,12), and only a few studies reported promising results in male soccer players after a more intensive training program compared with the one used in this study (19). As female players show different cutting execution (44,46) and need other resources to optimize learning (5) compared with male players, similar responses to training programs could not be expected. Second, learning complex tasks (i.e., unanticipated sidestep cutting) needs more information sources compared with simple tasks (31). Whereas others have found positive effects on improving movement execution in anticipated cutting (19,38,51), the complexity of unanticipated SSC in this study may ask for more information sources to find benefits on learning. Finally, we used a new, advanced analysis method which separates amplitude and temporal characteristics (42) and accounts for the model subjects had seen. It is difficult to compare this with previously used methods, and others may have had positive results that might not hold up when applying the current method.

The applied intervention included the characteristics found to be effective by Ste-Marie et al. (50); videos from peers from a posterior view with high model similarity (anthropometrics and sex) before and during training. While we implemented an optimized design, the supplement of the video instructions on top of the intervention itself did not have a high enough dose to provoke interaction effects. The expert videos were shown twice during each block of 10 trials every session, more research should be done to examine the appropriate frequency of providing video instructions. Possibly, video feedback is needed for one's own perception to optimize the motor learning curve (e.g., by showing expert videos and videos of the subject herself) (26,40). This may then be explained by greater neural activity when observing self-generated actions as opposed to observing other individuals' actions (26). Moreover, verbal feedback could be added, and previous research found beneficial effects in female athletes (5).

The individual baseline to retention differences shows clear distinction between subjects. Some subjects showed high baseline to retention differences and may have tightened the gap with the expert model, whereas others did not show large differences or even show negative differences (i.e., away from the expert values). In other words, high variability in response to the video instruction was observed. This is not surprising when it comes to response to injury prevention programs (15,20,23). Mixed responses to training intervention conceal potentially meaningful differences based on group mean analysis. This substantiates the importance of individual analyses instead of group analysis when assessing effectiveness of training programs (31,45,46).

To the best of our knowledge, this is the first study that investigated the effect of a long-term video intervention program instead of over short-time scales (i.e., 24 hours) in female soccer players. The inclusion of a retention test makes it possible to determine whether true motor learning occurred in this population (60). By contrast, others justified ACL injury prevention programs without measuring retention (24,29,37), which may have led to overestimation of their effectiveness on biomechanics. Therefore, future research should include retention tests to avoid false conclusions about effectiveness based on short-term effects as mentioned above. In addition, the effects of observational learning on movement execution of a complex, sport-specific skill were examined instead of relatively simple, discrete tasks (50). Moreover, to account for different heights and accompanying differences in limb movements (i.e., a taller person may make larger steps or jumps higher), 3 models were chosen as experts (1). Therefore, data of each subject were compared with the belonging expert based on height, and analyses were performed on the deviation waveforms. Finally, besides the upcoming number of studies using SPM mapping in biomechanics (62), this study used nonlinear registration to perform multivariate analysis of both amplitude and timing effects. This is important because amplitude and timing are generally associated with different biomechanical constructs and typical SPM results (of linearly registered data) can be ambiguous in terms of amplitude vs. timing effects. Amplitude is mostly associated with mechanical capacity (i.e., strength and loading magnitude), whereas timing is generally associated with coordination and control (i.e., neural activation patterns and kinematic style) (42). Although complete separation is not possible, the current analyses performed shed more light on the mechanism (i.e., amplitude of abduction moments) responsible for increased injury risk (42).

Although our sample size is not unusual within biomechanical research (12,19,51) and 1D sample size calculation was not possible, caution should be taken when generalizing our results to other populations or tasks. Moreover, this low sample precluded splitting the subjects up into responders and nonresponders and perform more detailed analysis. Only 10 to 15 (depending on the week) practice trials of SSC were performed during each training session. Speaking about the dose-response relationship (25), subjects probably would need more practice trials (dose) to improve their movement execution (response). The current design included training sessions with 3 tasks and a progression in load during the weeks to elicit a variety of movements all related to injury prevention. Therefore, it was not possible to increase the number of practice trials per task.

More research is necessary to examine which additional tools could be used to elicit the potential of video instruction in female athletes. This study showed that expert modeling alone was insufficient to improve movement execution, when looking at it at the group level. The logical follow-up study to the present one would be to increase the training frequency or add video feedback in addition to video instruction. The former one has proven to be effective in improving anticipated cutting (38) and jumping (13,56) in male athletes. It is interesting to explore if this combination is also beneficial for female athletes. Finally, this study investigated cutting movements in a laboratory setting, and constraints were added to make it sport-specific. However, more interventions should explore the potential of video instruction and feedback in on-field situations instead of in a laboratory (49,50).

This study demonstrated improvements in hip abduction angle and moment, and knee abduction moment taking all subjects together. The improvements found in the frontal hip moment during IC and weight acceptance implies that the program was effective in reducing biomechanical risk factors for ACL injury in the short-term. Large interindividual differences were found which may have concealed the effects of the video instruction at the group level. Additional instruction or feedback modes are possibly needed to optimize movement execution in female soccer players. Moreover, investigations should be undertaken into individual analyses instead of group analysis to determine the effectiveness of intervention programs.

**Practical Applications**

This study enhances our understanding about the use of video instruction in female soccer players to improve quality of movement. Providing video instruction solely twice during 10 practice trials is probably not enough. Therefore, video instruction has the potential to improve movement execution, but additive video feedback should certainly be considered when optimizing intervention programs. During practice, this could be done by showing athletes their movements captured with smartphones or tablets for example. This allows them to view how they personally perform the movement task and actively problem solve (by evaluating the mistakes and good points of their trials) to develop techniques and find individual ways to improve movement execution. In addition, coaches are advised to incorporate variation and sports specificity in their training programs to challenge athletes. For example, mixing unanticipated deceleration and cutting in different directions can be used in combination with adding (different types of) balls to hit a certain goal. Finally, our findings indicate that the effects of the current program may be dependent on an individual athlete. Athletes may learn differently and coaching should be adjusted accordingly, i.e., 1 athlete needs more feedback (resources) compared with someone else for whom only video instruction is sufficient. Finally, model similarity can be enhanced by using several expert models, and coaches are advised to use peer models with matching anthropometrics and sex.

## Supplementary Material
