# Supplementary material for: Observe, Practice, and Improve? Enhancing Sidestep Cutting Execution in Talented Female Soccer Players: A Four-Week Intervention Program With Video Instruction
Source: J Strength Cond Res. 2024 Apr 25;38(8):e430–9. doi: 10.1519/JSC.0000000000004796 (PMC11286158; doi:10.1519/JSC.0000000000004796)
Supplement: Supplementary file 1 [file jscr-38-e430-s001.docx]

SDC 1: Detailed description of tasks of the program.

1. **Unanticipated sidestep cutting**

*
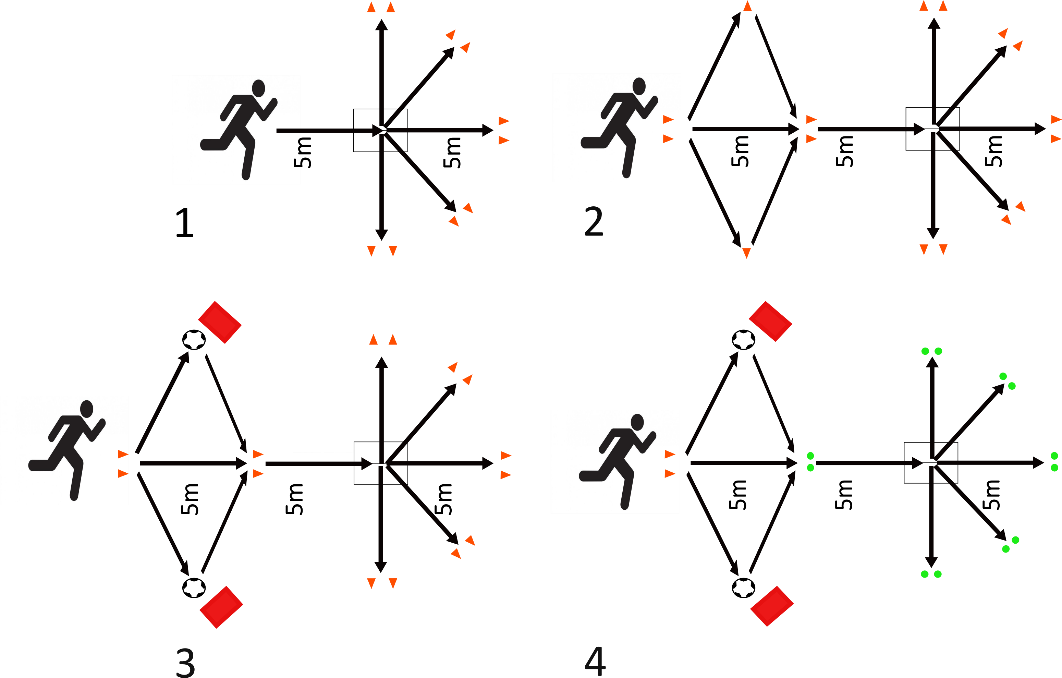
*

*Figure S.1 Sidestep cutting task.* *Explanation: the direction is determined by buddy. Buddy and athlete stay behind each other, the buddy starts to run and athlete has to respond visually. In week 3 and 4, the athlete must hit the ball to the target. In week 4, the cones are replaced with lights, one of the lights flashes up when passing the start gate.*

1. **Single leg jumping and landing**


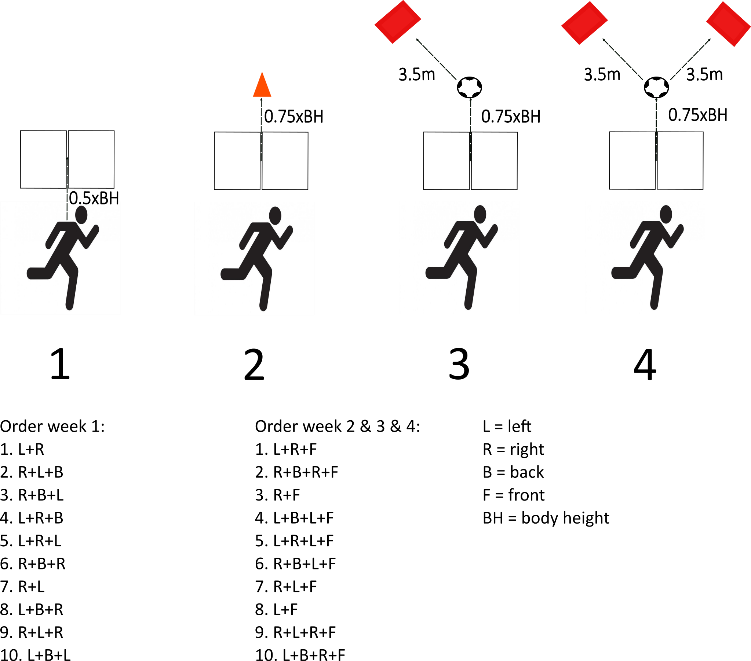


*Figure S.2 Single leg jumping and landing. Explanation: Buddy stands 5 meters in front of the athlete. Test leader counts down 3-2-1-go and buddy starts to jump, athlete follows. In week 2, athletes touches cone at last landing. In week 3 and 4, trial only counts if ball hits target. The target is left when shooting with right leg and vice versa (week 3). In week 4: buddy makes double leg jump after last single leg hop and indicates shooting direction when in the air (hand to the left or right)*

1. **Double leg jumping and landing**

**
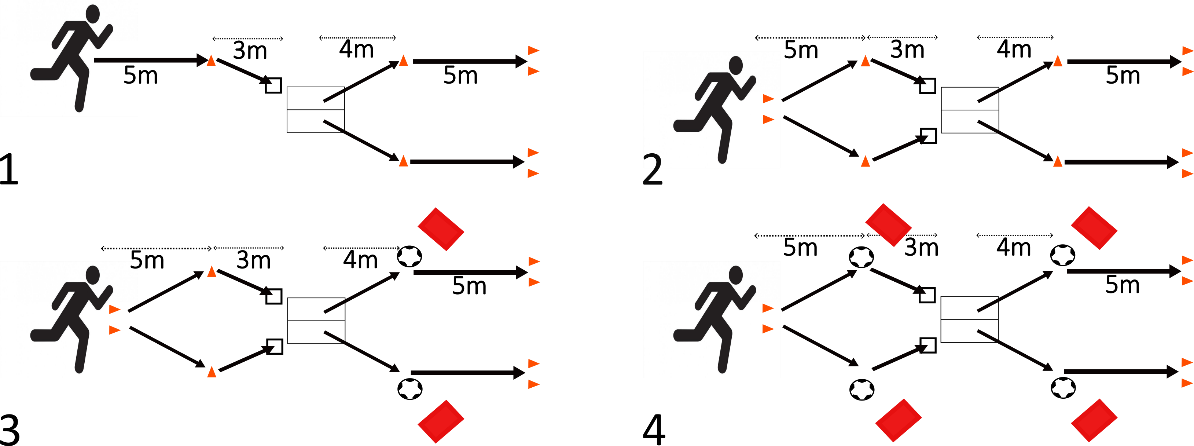
**

*Figure S.3 Double leg jumping and landing, note: figure is displayed for right dominant leg. Explanation: Buddy stands 1.5m in front of the athlete. Test leader counts down 3-2-1-go and buddy starts to run to the first cone (week 1) or shuffle to left or right (week 2-4). The athlete makes a half turn and follows buddy. From first cone, sprint to the small square and push off in small square with one leg and land on two legs on forceplates, immediately jump and land on two legs. Shuffle to left or right (determined by buddy) to the next cone and sprint to the finish. The distance between small square and mid forceplate is ½ body height of the athlete. Trial only counts if athlete approaches forceplates from left (if right leg dominant). In week 3 and 4, trial only counts if ball hits target(s).*
