# Supplementary material for: Observe, Practice, and Improve? Enhancing Sidestep Cutting Execution in Talented Female Soccer Players: A Four-Week Intervention Program With Video Instruction
Source: J Strength Cond Res. 2024 Apr 25;38(8):e430–9. doi: 10.1519/JSC.0000000000004796 (PMC11286158; doi:10.1519/JSC.0000000000004796)
Supplement: Supplementary file 2 [file jscr-38-e430-s002.docx]

SDC 2: Table with kinematic and kinetic values of chosen expert trials of sidestep cutting task.

Table S.1 Knee kinetics and kinematics of trials selected for video instruction

| Body height (cm) | Knee flexion angle(°) | Knee flexion moment (Nm/kg) | Knee varus(+)/valgus(-) moment (Nm/kg) | vGRF (N/kg) | Knee range of motion (°) |
| --- | --- | --- | --- | --- | --- |
| 165-170 | 53.8 | 1.30 | 1.35 | 15.50 | 25.9 |
| 170-175 | 52.6 | 2.43 | 1.64 | 14.50 | 38.6 |
| 175-180 | 63.1 | 1.47 | 1.25 | 17.90 | 43.2 |
| Except of knee range of motion, all variables were determined at timeframe of peak varus/valgus moment during IC (0-40ms). Knee range of motion was calculated using the difference in angle from t=0 to maximum knee flexion. | | | | | |
