# Supplementary material for: Observe, Practice, and Improve? Enhancing Sidestep Cutting Execution in Talented Female Soccer Players: A Four-Week Intervention Program With Video Instruction
Source: J Strength Cond Res. 2024 Apr 25;38(8):e430–9. doi: 10.1519/JSC.0000000000004796 (PMC11286158; doi:10.1519/JSC.0000000000004796)
Supplement: Supplementary file 3 [file jscr-38-e430-s003.docx]

SDC 3: Figure with individual baseline values of each variable


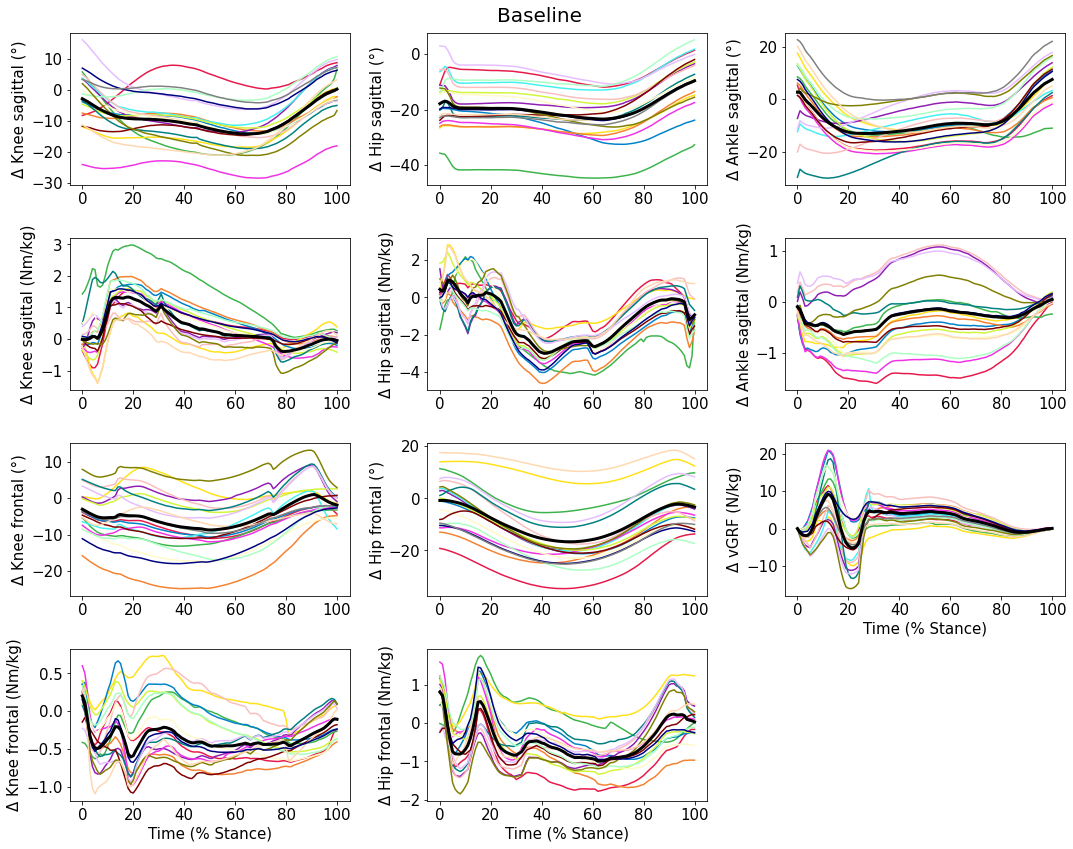


*Figure S.4 Individual baseline differences between subject and expert of all variables. Greater values indicate higher values of subject compared to expert. Each colour represents one subject. The black line indicates the mean change from baseline to retention.*
